# Psychiatry out-of-hours: a focus group study of GPs' experiences in Norwegian casualty clinics

**DOI:** 10.1186/1472-6963-11-132

**Published:** 2011-05-27

**Authors:** Ingrid H Johansen, Benedicte Carlsen, Steinar Hunskaar

**Affiliations:** 1 National Centre for Emergency Primary Health Care, Uni Health, Kalfarveien 31, Bergen, Norway; 2 Department of Public Health and Primary Health Care, University of Bergen, Kalfarveien 31, Bergen, Norway; 3 Uni Rokkan, Nygaardsgt. 5, Bergen, Norway

## Abstract

**Background:**

For Norwegian general practitioners (GPs), acute treatment of mental illness and substance abuse are among the most commonly experienced emergency situations in out-of-hours primary healthcare. The largest share of acute referrals to emergency psychiatric wards occurs out-of-hours, and out-of-hours services are responsible for a disproportionately high share of compulsory referrals. Concerns exist regarding the quality of mental healthcare provided in the out-of-hours setting. The aim of this study was to explore which challenges GPs experience when providing emergency care out-of-hours to patients presenting problems related to mental illness or substance abuse.

**Methods:**

We conducted a qualitative study based on two individual interviews and six focus groups with purposively sampled GPs (totally 45 participants). The interviews were analysed successively in an editing style, using a thematic approach based on methodological descriptions by Charmaz and Malterud.

**Results:**

Safety and uncertainty were the dominating themes in the discussions. The threat to personal safety due to unpredictable patient behaviour was a central concern, and present security precautions in the out-of-hours services were questioned. The GPs expressed high levels of uncertainty in their work with patients presenting problems related to mental illness or substance abuse. The complexity of the problems presented, shortage of time, limited access to reliable information and limited range of interventions available during out-of-hours contributed to this uncertainty. Perceived access to second opinion seemed to have a major impact on subjectively experienced work stress.

**Conclusions:**

The GPs experienced out-of-hours psychiatry as a field with high levels of uncertainty and limited support to help them meet the experienced challenges. This might influence the quality of care provided. If the current organisation of emergency mental healthcare is to be kept, we need to provide GPs with a better support framework out-of-hours.

## Background

In countries with two-tier public healthcare systems there is a debate over how to best provide emergency psychiatric care [1,2]. Some countries, like Norway, adhere to a system where general practitioners (GPs) are gatekeepers for all specialised care [3,4]. Thus in Norway regular general practitioners (RGPs) provide emergency psychiatric care for their enlisted patients during office-hours [5], and they are responsible for onwards referral of patients in need of emergency psychiatric care at a more specialised level. Out-of-hours, emergency primary healthcare is the responsibility of local municipalities [6]. This requirement is normally met by organising a casualty clinic with one or several GPs on duty. For the most part, the GPs on call are RGPs working in the same municipality [7]. Depending mainly on the size of the population served, these GPs might work alone or be supported by other health personnel [3]. Some of the largest casualty clinics are open around-the-clock, providing daytime emergency care for patients who cannot access their RGP. However, in this article we will use *casualty clinics *and *out-of-hours services *interchangeably.

Despite the fact that a rather low rate of mental illness is presented out-of-hours in Norway [8,9], nearly all GPs working out-of-hours are exposed to emergency psychiatry in the course of a year [10]. Out-of-hours psychiatry differs from daytime psychiatry by consisting of relatively more serious mental illness, such as suicidal behaviour, psychosis and substance misuse [11]. The GPs are thus likely to meet more severe diagnostic challenges out-of-hours than during daytime practice.

Previous research on casualty clinics in Norway have used the chapter P of the diagnostic system International Classification for Primary Care 2^nd ^edition [12] to identify cases of mental illness [8,11]. This chapter includes psychological symptoms and complaints, psychiatric diagnoses and substance misuse diagnoses. Addiction treatment has been part of the medical system in Norway since 2004. The co-morbidity between substance abuse and mental illness is high. Approximately a third of patients admitted to emergency psychiatric wards in Norway have a substance use disorder [13]. Many of these patients are referred from casualty clinics.

In Norway there are 20 times more consultations related to mental illness by RGPs in their daytime practice compared to GPs at casualty clinics [9]. Still, studies have shown that the largest share of acute referrals to emergency psychiatric wards comes from casualty clinics [14,15]. Casualty clinics are also over represented as referring agent when compulsory psychiatric care is involved [15]. Thus there are concerns regarding RGPs limited role in emergency psychiatric care for their enlisted patients and the quality of psychiatric assessments made at casualty clinics [16]. It is popularly presumed in Norway that GPs perform worse at casualty clinics compared to when they work as RGPs. So far, this has very limited scientific support [17]. However, in informal discussions GPs often express some problematic issues regarding psychiatric patients at casualty clinics. Existing qualitative research on psychiatry in primary healthcare has mostly focused on daytime general practice [18-23]. To our knowledge, no former study has focused on the challenges GPs meet out-of-hours. In this study we therefore wanted to explore the GPs' experiences of dealing with casualty clinic patients presenting mental illness or substance abuse, hoping that these experiences could inform possible ways of improving the out-of-hours services for this patient group.

## Methods

Due to the exploratory purpose of this study, we chose a qualitative design. We performed focus groups to reduce the impact of the interviewer and to enhance memory retrieval and sharing of views between informants. We also performed individual interviews with two informants who agreed to participate in the study, but who for practical reasons could not participate in any of the group discussions.

### Participants

The participants were purposively sampled in order to represent both genders and to include informants of varied age and length of work experience. We actively recruited GPs with experience from differently organised casualty clinics. Thus the interviews included GPs working alone when on call, GPs working at casualty clinics where one GP is on call and is supported by other health personnel, and GPs working at large casualty clinics with several GPs and other health personnel on duty.

Initially we attempted to recruit the focus group participants individually. However, this strategy only recruited GPs who were particularly interested in psychiatry (individual interview 1+2). To include a broader range of GPs, we invited pre-established peer groups of physicians specialising in general practice to participate. Participation in a supervised peer group with regularly held meetings over a two year period is compulsory for all physicians specialising in general practice in Norway. These groups are organised by the Norwegian Medical Association. We contacted peer groups in regions of Norway with differing degrees of co-organisation between specialist care for psychiatry and specialist services for substance abuse treatment. The peer groups were approached through their supervisors. All approached groups agreed to participate. Due to it's size (12 members and 2 supervisors), one of the peer groups was divided in two for the group discussion [24], thus creating one focus group with mainly younger GPs (focus group F), and one with the supervisors and the most experienced GPs (focus group E). At the time of interview the peer groups had gathered for regular meetings from 2 until 22 months.

To ensure participation of GPs working in large city-based casualty clinics, the administrator of one of the largest casualty clinics in Norway was approached. She recruited participants for one focus group (focus group B). The administrator was not present during the interview.

In total, the study consisted of 2 individual interviews and 6 focus groups. Table 1 gives an overview of the groups. In the third and forth group no new themes emerged. Still, we decided to carry on with the interviews because another two groups were already recruited and differed from the previous groups in terms of composition of the group (focus groups E and F). Although no new themes emerged in these last groups, they gave important nuances to the description of the already identified themes.

**Table 1:** Overview of the focus groups A-F (n = 6).

	Group participants					Experiences present in the group regarding organisation of casualty clinic
		
	Total	Men	Women	Age (range)	Years of experience	
**A**	7	4	3	29-53	3-22	One GP on duty with or without support by other health professionals
**B**	6	3	3	31-33	2-4	Large casualty clinic with several GPs on duty
**C**	7	2	5	30-57	2-22	One GP on duty with or without support by other health professionals
**D**	9	2	7	32-54	2-25	All types
**E**	7	3	4	32-55	2-23	All types
**F**	7	2	5	30-34	1-2	Large casualty clinic with several GPs on duty

### Data gathering

The interviews were conducted by BC (focus group E) and IHJ (all other interviews) in the period between February and November 2009. All interviews were recorded by digital sound-recorder. The individual interviews lasted 40-44 minutes. The focus groups lasted 81-113 minutes. Most interviews were conducted at the location where the peer groups normally gathered for supervision. The other interviews were conducted at IHJ's workplace (individual interview 1), and the participants' workplace (individual interview 2 and focus group B).

The participants were encouraged to talk freely, and the interviews were structured around the following three topics:

- An out-of-hours consultation or home visit where the patient presented mental illness or substance misuse

- An out-of-hours consultation or home-visit where the patient presented mental illness or substance misuse and where the consultation or home-visit had an unexpected positive turn

- Suggestions for how to improve the working conditions for GPs when dealing with patients presenting mental illness or substance misuse

Towards the end of the interview the participants were encouraged to share thoughts they had had during the interview which had not been covered in the discussion. They were also asked if there was something they wanted the researchers to know about their experience of psychiatric patients at casualty clinics which had not been addressed during the interview.

### Analysis

The analysis was performed in an editing style [25]. The actual process was based on methodological descriptions by Charmaz [26] and Malterud [27]. Each interview was transcribed verbatim by IHJ. The transcript was then read by IHJ (GP) and BC (social anthropologist), identifying themes independently. Preliminary results were discussed. The interviews were spread out in time, and the preliminary analysis of each interview was carried out shortly after the interview. In successive focus groups preliminary results were actively challenged by searching for discordant experiences and views. In some of the later groups, preliminary results were presented at the end of the group session when time allowed, and the participants were then asked to comment on them. After the initial analysis of the last transcript, IHJ and BC agreed on a framework for coding. IHJ coded all transcripts. Some sections were control-coded by BC. Presence or absence of text related to codes in each transcript was recorded in a matrix.

A hierarchy of principal and subordinate themes was built based on further analysis of text related to each code. The descriptions of the themes were evolved through writing memos. The memos were refined in constant comparison with the text, and they later constituted the basis for presenting the results. Quotations were selected to show illustrative and typical parts of the transcripts. The interviews, the transcription and the initial analysis were conducted in Norwegian. The memos were written in English. The selected quotations from the transcripts were translated into English, and then re-translated into Norwegian to secure identical meaning of the statements.

### Ethics

The project was approved by the Norwegian Social Science Data Services. The Regional Committee for Medical Research Ethics had no objections to the study. All participants received written and oral information about the study and gave written informed consent to the moderator of the focus group. There was no payment involved.

## Results

The group discussions were animated. In most groups the participants discussed between themselves with minimal interruption of the moderator. In general, the participants had similar experiences of dealing with casualty clinic patients presenting mental illness or substance abuse, but occasional outbursts of open disagreement occurred. These outbursts tended to be more frequent and insistent in peer groups with a longer history of meetings. Disagreements between group participants will be specifically addressed in relation to the relevant themes. Two principal themes emerged from the analysis: Safety and uncertainty.

### Safety

The theme personal safety arose spontaneously in all the interviews. None of the GPs described psychiatric patients as dangerous in general. However, the GPs worried about unpredictable behaviour, especially when the patient appeared intoxicated, displayed drug seeking behaviour or the GP needed to inflict compulsory care on the patient. Not knowing the patient from before increased the GPs' concern about predicting the patients' behaviour correctly. Most of the GPs had been threatened verbally by patients or the patient's relatives. Really dangerous situations were considered rare, but several of the GPs narrated stories about situations which had suddenly and unexpectedly taken a nasty turn and where they had felt themselves physically threatened, sometimes life-threateningly. When finding themselves in a threatening situation some of them had contacted other health personnel or the police to get help and reinforcement without being able to mobilise the needed support. Some of the GPs reflected on their reduced ability to do a good job in situations where they felt anxious, as their focus were then shifted to concerns over own security instead of being with the patient and the presented problem.

Some of the GPs described themselves as relatively naïve regarding security matters. They seemed to genuinely believe that their status as health personnel and helpers protected them in dangerous situations. They described how in actual situations they were absorbed by their duty as health personnel and by medical issues, without paying attention to their own security. However, considering episodes retrospectively they sometimes felt themselves lucky to have survived, or at least, to not have been injured. Several of the GPs said that they had become more precautious after the experience of a dangerous situation, and a few of the GPs described a sustained uneasiness before consultations and home-visits if they knew the patient had a mental illness or an addictive disorder.

'GP 2: I don't like driving duties. I don't mind doing shifts at the casualty clinic, in the daytime or the evening or at night. But it's having driving duties, having to drive around and not knowing exactly what you are coming up against. Things can seem okay on the phone, and then you go out and it's not okay at all. And you might have a driver with you, and that's all. I don't think that's much fun.

GP 3: When I did my placement, I was completely alone when I was on call. There was no nurse or anything. And then too, to get up in the middle of the night and drive around by yourself. That wasn't much fun. I always had this rule: First try to survive. That was my aim for the shift. And then there was to not kill anyone. And then to do a good job was number three. But it's always like this, I always felt after the shift that it had been fine. But I was always worried beforehand. Always uneasy before, because you don't know what will happen.

GP 4: You always have a high level of tension before you go on duty at the casualty clinic.

(.......)

GP 5: I feel that other emergency situations are stressful because I don't do them too often. But this is different. It's about your security. So if I had felt certain that it was completely safe, I don't think I would have worried in the same way.'

(Focus group F)

To be alone and completely left to your own device was a common theme, and for many of the GPs this was the most unpleasant part of working at casualty clinics. The presence of others, either other health personnel or police, was seen as a reassurance. Many of the GPs questioned the routine security measures and awareness at the casualty clinics, for example when carrying out unaccompanied home-visits or when the consultation rooms were arranged without any escape routes or possibilities to alert others if a dangerous situation arose. At the same time as the GPs called for higher levels of safety, most of them were strong advocates for the strategy of defusing situations by meeting patients face-to-face and without extensive security precautions. They claimed that extended security measures could be interpreted as a display of distrust, and this conflicted with the GPs' common philosophy of trusting patients. The focus group participants often had dissension regarding the appropriate standard level of precautions, and some GPs claimed that high security measures directed towards individual patients could escalate the problems and prevent a helpful dialogue. Some GPs also argued that standard warnings could unwarrantedly prevent or delay patients in receiving necessary healthcare. The issue of precautions for their own security versus consideration for the patient's dignity was raised as an ethical and practical dilemma. For example, most groups discussed the stigmatising effect of doing home-visits accompanied by police, and how that had to be balanced against their own need of feeling confident and at ease.

### Uncertainty

Uncertainty also emerged spontaneously as a theme in all the interviews. The uncertainty described consisted of a perceived complexity of the issues presented, suboptimal preconditions for the patient encounter and factors related to the GPs' personal confidence.

#### The complexity of the presented issues

The presented issues were sometimes perceived to be complex and not just about psychiatry. The GPs talked about cases they had seen at the casualty clinic which were not necessarily psychiatry, but which clearly did not fit into any other medical category. Examples were situations where patients presented symptoms which did not correspond to descriptions of recognized medical syndromes, situations where the patient had displayed behaviour disturbing to people in the surrounding environment without this behaviour necessarily implying a severe mental illness, and situations where the patient obviously had a severe mental illness, but the presented and pressing issue was of a different character, for example housing problems. The GPs talked about how they were approached by the patients themselves, or by the patients' relatives or health personnel on the behalf of patients, with an expectation that they would solve problems which did not relate to their medical expertise. Some of the GPs argued for the GPs' legitimacy of not accepting responsibility for these problems. On the contrary, some of them argued that the act of contacting a casualty clinic often reflected a desperate need for help from the parts involved, and that they as GPs had a strong obligation to help whatever the matter.

#### Preconditions for the encounter with the mentally ill patient

The GPs raised several problem areas in the encounter with the psychiatric patient which, although mentioned by all, were problematic to differing degrees in differently organised casualty clinics. First, the GPs talked about lack of information. At the casualty clinic they often had limited, if any, personal knowledge of the patient and there was no existing treatment alliance. They generally reported a lack of information about the patient's psychiatric history, present situation, and follow up. The GPs thus often felt completely reliant on the information the patient could provide. In cases with severe mental illness, intoxication or language barriers, such information could be uncertain or insufficient. The GPs said that they rarely had access to former assessments from psychiatric specialists, and that they seldom had access to updated information from daytime primary healthcare services. Thus they often felt that they had a faltering basis for their assessments.

Second, the GPs pointed to undisturbed time as a key premise for decent care of patients presenting problems related to mental illness or substance abuse. These patients were expected to need extra consultation time, and the logistics following the treatment decision was often even more time-consuming than the actual interaction with the patient. In contrast to their experiences with most other patients, the GPs sometimes encountered major obstacles when trying to arrange hospitalisation or even transport to the hospital. Most GPs expressed that they had a high workload when on duty, and they described a practical and emotional need of completing one consultation before moving on to the next patient. The use of extra time for one patient had consequences for other patients already waiting for the GP's services, and the GPs were stressed by the awareness of keeping other patients waiting. At clinics whose organisation meant that other waiting patients were not an issue, GPs still said that they sometimes had problems concentrating sufficiently during the patient encounter due to a permanent awareness of the possibility of an additional and simultaneous emergency call-out.

'GP 7: And what I also find challenging in a way, or even a bit scary, is the time pressure one is under at the casualty clinic. And you have minimal information to guide you. And it is then, when nobody around you will take responsibility, and the relatives say that they won't dare take him [the patient] home, and the registrar at the psychiatric hospital says that no, we don't want him. And, so you feel very alone.

GP 3: And there is no possibility of following it up.

GP 7: No.

GP 3: It is very different if you see someone during daytime. Then what you can say is 'ok, I'll call you tomorrow, and then we'll see how it's going'.'

(Focus group D)

Third, the GPs talked about how the available interventions did not correspond to the complexity and variety of the problems that some of the psychiatric patients presented. Most patients could be helped by the GP alone through counselling, prescribing medication or providing sick-leave. However, as soon as the presented problem could not be resolved by the GP alone, the GPs were frustrated by a 'limited toolbox', often described as consisting of an emergency admission or 'nothing'. When trying to help the patient out-of-hours, the GPs ran into practical barriers like difficulties in mobilising the necessary help from other services. The GPs advertised the need of a 'postponement-tool' for patients not necessarily in need of an immediate admission to a psychiatric hospital, but definitely in need of more than the GP could provide during a consultation. Examples of suggested 'postponement-tools' were direct follow up by community healthcare nurses, a safe place for the patient to sleep overnight, or a secured arrangement for follow up at the appropriate level of care the next day.

Independent of psychiatric training and personal views of the speciality, all the GPs expressed a need for consulting with a psychiatrist when in doubt about treatment decisions. Most of them lacked this option and were utterly frustrated with what they described as low service-mindedness in psychiatry compared with other medical specialities. The emotionality in these discussions was rather overwhelming. Surprisingly, the GPs' narratives of consultations or home-visits with an unexpected positive turn were mostly situations where the interaction with specialist services in psychiatry went smoothly, or where the GP unexpectedly managed to resolve the presented problem without the need of further support. Collaboration with the specialist services in psychiatry was often described as more challenging than the patient encounter, and several GPs said that they dreaded to call the psychiatrists. In four out of six focus groups the problematic interaction with specialist services in psychiatry seemed to completely overshadow other challenges in the encounter with psychiatric patients. The last two groups (focus group E and F) told of an unproblematic interaction with the specialist psychiatric services. These GPs described access to accommodating specialist advice around-the-clock. They said that the experience of the psychiatrists' availability and accommodating attitude constituted a major improvement in their working conditions.

'GP 8: But now it's a lot easier because we get help to do it [the assessments]. So I feel it's no longer a big load.

GP 1: There has been a marked change of culture here. And this happened some years ago. They also made some organisational changes regarding admittance, and created a ward for emergency admittance. And what that did - there were surely some changes in professional attitude in there as well - but in any case the result was what you're describing, that it became a lot easier to get contact [with the psychiatric specialists], a lot easier to get advice, and, not least, easier to get a second opinion or a treatment strategy when substance abuse was involved. That was just a big 'no' before. If they [the patients] were drunk and suicidal, then it was...

GP 7: The medical ward or nothing.

GP 1: ...futile [to contact the psychiatric specialists].

GP 8: It was good to have the police taking them for detoxification.'

(Focus group E)

Fourth, the GPs talked about patients that nobody wanted to take responsibility for, and who they sometimes failed to refer to a higher level of healthcare. Examples of this were patients with chronic suicidality, patients with personality disorders, patients with substance abuse with or without accompanying psychiatric problems, and patients displaying aggressive behaviour. The GPs reported that some of the otherwise available services refused to accept intoxicated patients. In such instances the GPs said that they had to use their creativity to find other and less desirable solutions for the patients, like leaving intoxicated patients in police custody till the patients' level of intoxication decreased.

#### Personal confidence

The GPs talked about how they often felt unsure about their final decision. They missed getting feedback on the decisions they had made. Such feedback was considered important to improve their own practice, and also to assure them that they had done the right thing. Many of the GPs claimed they felt an emotional and practical barrier to admitting psychiatric patients. Emergency admissions, especially when involving compulsory care, were described as a last resort when they saw no other obvious solution. As many of them felt badly about admitting patients to emergency psychiatric wards, they often sought reassurance that the patient benefitted from being admitted. Many of them described relief when their decisions were approved by the specialists.

The GPs also talked about the fear of not making the right decision when assessing suicide or danger risk given the dire consequences of a mistake - e.g. compared to a mistake in diagnosing for example stomach pain. Many of the GPs reported brooding over the patient encounter or poor sleep quality after having failed to secure acute follow up for a chronic suicidal patient or a person with serious substance abuse, or after having referred a patient to compulsory care. For some of the GPs the experienced uncertainty when handling psychiatric cases was so pronounced that they would prefer to not have to deal with psychiatry out-of-hours at all, at least not under the present conditions.

'GP 1: ...we feel apprehensive about getting psychiatry and substance abuse when we're on duty. I'd rather have five myocardial infarctions than one psychiatric case. That's what I think anyway.

**IHJ: And this is due to the factors you have discussed already, that it takes time and that you don't know what to do**...

GP 1: Yes. Uncertainty, lack of information, and uncertainty over the right diagnosis, and worry over whether you'll be understood at the hospital, and all these things.

GP 6: Yes. It's a messy field in a way. That's always, that's what we really have been talking about the whole time, all this mess. These kinds of cases don't go smoothly.

GP 4: But I do think there's great potential for making them go more smoothly. I think there are many ways of making it better.'

(Focus group A)

## Discussion

This study suggests that Norwegian GPs experience out-of-hours psychiatry as a field with high levels of uncertainty and limited support to help them meet the experienced challenges. Despite wide consensus across the focus groups and the inclusion of GPs with varied levels of experience and from different out-of-hours settings, the applied methodology limits our ability to assess the magnitude of the identified problems. The findings thus need further validation by triangulation, for example by survey-data from a more representative group of GPs or by observational studies of consultations and home-visits. By the end of the interviews the informants often stressed that they had spent most of the interview delving into problematic issues, suggesting we might have elicited an unwarranted grim impression of how GPs view their working conditions. However, even if the reported problems are marginal in magnitude, they still seemed to strongly colour these GPs' experiences with cases of mental illness or substance misuse. There is reason for concern that the GPs' work experiences might have a negative impact on the interaction with patients and other parts of the healthcare services.

Qualitative interviews are dynamic processes [28], and research has shown that interviewing professional peers creates special issues like the informants experiencing the interview as a test of knowledge, competence or even morale [29]. In this study these issues were not apparent apart from in one of the individual interviews. The use of established peer groups could be argued to limit the usefulness of the data collected as participants might make their narratives more socially acceptable to preserve relationships within the group. This is, however, a general weakness of group discussions, and can only be entirely avoided by choosing a different design. In this study established power constellations and group confidentiality seemed to encourage the sharing of very personal experiences and views, including open disagreement between participants. Groups with a shorter history together tended to spend more discussion time testing the social acceptability of their statements. Nevertheless, it is likely that performing individual interviews could have generated information inaccessible under the current design. However, our problems in individual recruitment suggest that such an approach might have been even more problematic due to selection bias of the interviewees. By using established peer groups we managed to elicit some voices of GPs with low interest in psychiatry, and although their statements might have been modified, they could still balance the view of more positive GPs.

The main moderator (IHJ) had the same professional background as the focus group participants, and it could be argued that this could decrease the level of detail in the data and preclude the necessary distance in the analysis. The focus group design was chosen specifically to reduce the impact of the moderator, and in the execution of the study we saw a tendency to more vivid inter-participant discussions and thicker descriptions in the groups moderated by the GP compared to the group moderated by the social anthropologist. It might be that sharing professional background was an advantage in the collection of data. However, group dynamics differ, and the observed difference could be unrelated to the moderator. The joint analysis by a junior GP, a social anthropologist and a senior GP and professor (SH) was important in reducing the influence of the different researchers' preconceptions on the analysis. The different viewpoints of the social anthropologist proved especially valuable in unveiling blind spots and ensuring enough distance to the produced data.

Our findings are in accordance with findings from other studies in general practice. The concern regarding personal safety has previously been reported in qualitative studies on GPs' experiences of violence in UK and Australia [30,31]. One of these studies was conducted 20 years ago [30], indicating that personal safety is no new worry for GPs. Emergency medicine and psychiatry have been singled out as high risk areas for experiencing work-related violence [32,33]. Reported prevalence of experienced violence among GPs ranges from 21% to 83% depending on methodology and geographical area studied [34-41]. The apprehension about work-related violence has been found to be particularly high in out-of-hours services [31,42-44]. As most papers on work-related violence in general practice have pointed out, this might have serious consequences for a viable after-hours primary healthcare service. Ensuring GPs personal safety in out-of-hours services might increase the GPs' job satisfaction and contribute to fewer GPs opting out of the services. However, when increasing the personal safety for GPs, attention should be paid to the dilemma raised in this study between personal security and patient dignity [45], by applying measures that increases the GPs safety without stigmatising the patient or undermining the necessary atmosphere of trust in the doctor-patient relationship. Examples of such measures could be to have nurses or ambulance personnel accompanying the GP on home-visits, to have other health personnel present at the casualty clinic around-the-clock, to arrange consultation rooms so that the GP has an emergency escape route and to provide GPs with security alarms. If such measures are implemented, further research should be undertaken to explore the effects on prevention of serious consequences of dangerous episodes and to assess changes in the GPs' experiences of their working conditions and the effects on recruitment to out-of-hours services.

Uncertainty was the other core theme in our study, and dealing with uncertainty is a recognised challenge in medicine [46,47]. Beresford claimed that uncertainty is an inescapable element of the interpersonal, context-specific and judgement-dependent nature of medical practice [46]. He identified three sources of uncertainty: *technical*, *personal *and *conceptual *[46]. The technical source of uncertainty was described as the lack of adequate data to predict progress of disease or outcomes of certain interventions. The personal source of uncertainty was ascribed to the physician-patient relationship, i.e. the physician's ability to make a detached clinical decision and the problem of having limited information of the patients' preferences. The conceptual source of uncertainty was the problem of prioritising patients and the difficulty in applying abstract criteria to concrete, and often complex, situations. According to our study these three sources of uncertainty seemed to be present in out-of-hours general practise to an even higher degree for patients presenting problems related to psychiatry or substance misuse compared to other patients. In particular, the presence of substance abuse related problems seemed to complicate the GPs' work situation and amplify the reported levels of uncertainty.

Complex situations with limited information, limited knowledge of the patient and limited time seemed to force the GPs to make uncertain decisions on a faltering basis. They sometimes seemed to feel inadequate, and they sometimes raged at other parts of the system for letting them down. Especially, the emotional force in the discussions about the interaction with specialist services in psychiatry was rather overwhelming. The relation between specialists and generalists has been shown to be asymmetric [48], and it is acknowledged that GPs highly value access to second opinion [18,23]. One study has questioned the consensus between specialists and GPs over which patients to refer to specialist services, finding that GPs tend to refer when they have reached their subjective limit of competence [19]. Given the reported level of experienced uncertainty in our study, it is understandable that frustration and aggression are created by the perceived withholding of needed assistance in decision making and by the lack of shared responsibility for the patients. The 'limited toolbox' probably also fuels this frustration, especially as the absence of 'postponement tools' forces the GPs to acutely refer patients who they believe should rather be handled in other, but presently unavailable, ways. Thus the GPs find themselves trapped in a scapegoat position as either the doctor who refers patients unnecessarily or the doctor who prevents the patient from receiving required help.

Uncertainty in itself might lead to suboptimal care [49]. The individual tolerance of uncertainty varies [50], and it has been shown that uncertainty leads to anxiety and concern about bad outcomes [51]. Concern about bad outcomes is associated with burn-out [52], and the concern seems to lessen with increased work experience [53]. By contrast, it seems that experience does not lessen anxiety due to uncertainty [53]. Aversion to uncertainty is associated with a negative orientation against psychological problems, and predicts negative attitudes towards hypochondriac, geriatric and chronic pain patients [50]. Interestingly, all these patient groups rank low in medical hierarchies [54,55]. The position in a hierarchy could possibly affect the quality of acute treatment when the same emergency service is supposed to cater for all somatic and psychiatric conditions. An example of this is the finding that those working in the emergency room in a general hospital expressed less positive attitudes towards suicide attempters compared to those working in the emergency room at the psychiatric hospital [56]. Although the general atmosphere of discussion in our study differed among the focus groups, it was rare to find openly expressed negative attitudes towards patients presenting problems related to mental illness. The study rather pointed to structural and organisational barriers to optimal treatment. Of course, these barriers could reflect institutionalised discrimination [57]. All the same, interventions addressing attitudes only will have limited effect unless the structural shortcomings are amended. To ensure that the patients receive the best care possible, it is important that we organise our emergency healthcare system so that GPs have access to the necessary support in decision making independent of their personal work experience and tolerance of uncertainty.

## Conclusions

The expressed levels of insecurity and uncertainty when dealing with casualty clinic patients presenting mental illness or substance misuse suggest that many GPs lack adequate support in the provision of out-of-hours emergency mental healthcare. This might have implications for the quality of care provided. If the current structure of emergency psychiatric healthcare is to be kept, we must consider empowering the GPs by giving them a more appropriate support framework for interventions in mental healthcare. This support framework could consist of access to the necessary support in decision making, real alternatives to emergency admissions and better personal safety. Such initiatives could improve the GPs' work conditions, and probably also contribute to improved quality of care. However, implementation of changes needs scientific evaluation, and the connection between GPs' experienced work situation and the quality of care provided needs further investigation.

## List of abbrevations used

GP: general practitioner; RGP: regular general practitioner

## Competing interests

The authors declare that they have no competing interests.

## Authors' contributions

IHJ conceived of the study, participated in its design, performed the individual interviews, moderated five focus groups, analyzed the data and drafted the article. BC participated in the design of the study, moderated one focus group, contributed to the analysis of the data and revised the manuscript critically for important intellectual content. SH participated in the design of the study, gave input to the analysis and revised the manuscript critically for important intellectual content. All authors read and approved the final manuscript.

## Pre-publication history

The pre-publication history for this paper can be accessed here:

http://www.biomedcentral.com/1472-6963/11/132/prepub
